# Significant variance in genetic diversity among populations of *Schistosoma haematobium* detected using microsatellite DNA loci from a genome-wide database

**DOI:** 10.1186/1756-3305-6-300

**Published:** 2013-10-17

**Authors:** Travis C Glenn, Stacey L Lance, Anna M McKee, Bonnie L Webster, Aidan M Emery, Adhemar Zerlotini, Guilherme Oliveira, David Rollinson, Brant C Faircloth

**Affiliations:** 1 Department of Environmental Health Science, University of Georgia, Athens 30602 GA, USA; 2 Savannah River Ecology Laboratory, University of Georgia, Drawer E, Aiken 29802 SC, USA; 3 Warnell School of Forestry and Natural Resources, University of Georgia, Athens 30602 GA, USA; 4 Department of Life Sciences, Natural History Museum, Wolfson Wellcome Biomedical Laboratories, Cromwell Road, London, SW7 5BD, UK; 5 Rene Rachou Research Center, National Institute of Science and Technology in Tropical Diseases, Oswaldo Cruz Foundation, Av. Augusto de Lima 1715, BarroPreto, Belo Horizonte CEP 30190-002 MG, Brazil; 6 Department of Ecology and Evolutionary Biology, University of California, Los Angeles 90095 CA, USA; 7 Present address: Embrapa Agricultural Informatics, Av. Andre Tosello, 209, Campinas 13083-886 SP, Brazil; 8 Present address; Department of Infectious Disease Epidemiology, Imperial College Faculty of Medicine (St Mary’s Campus), Norfolk Place, London W2 1PG, UK

**Keywords:** Africa, Differentiation, F_ST_, Genomic, Microsatellites, Primer database, *Schistosoma haematobium*, Urogenital schistosomiasis

## Abstract

**Background:**

Urogenital schistosomiasis caused by *Schistosoma haematobium* is widely distributed across Africa and is increasingly being targeted for control. Genome sequences and population genetic parameters can give insight into the potential for population- or species-level drug resistance. Microsatellite DNA loci are genetic markers in wide use by *Schistosoma* researchers, but there are few primers available for *S. haematobium*.

**Methods:**

We sequenced 1,058,114 random DNA fragments from clonal cercariae collected from a snail infected with a single *Schistosoma haematobium* miracidium. We assembled and aligned the *S. haematobium* sequences to the genomes of *S. mansoni* and *S. japonicum*, identifying microsatellite DNA loci across all three species and designing primers to amplify the loci in *S. haematobium*. To validate our primers, we screened 32 randomly selected primer pairs with population samples of *S. haematobium*.

**Results:**

We designed >13,790 primer pairs to amplify unique microsatellite loci in *S. haematobium*, (available at http://www.cebio.org/projetos/schistosoma-haematobium-genome). The three *Schistosoma* genomes contained similar overall frequencies of microsatellites, but the frequency and length distributions of specific motifs differed among species. We identified 15 primer pairs that amplified consistently and were easily scored. We genotyped these 15 loci in *S. haematobium* individuals from six locations: Zanzibar had the highest levels of diversity; Malawi, Mauritius, Nigeria, and Senegal were nearly as diverse; but the sample from South Africa was much less diverse.

**Conclusions:**

About half of the primers in the database of *Schistosoma haematobium* microsatellite DNA loci should yield amplifiable and easily scored polymorphic markers, thus providing thousands of potential markers. Sequence conservation among *S. haematobium, S. japonicum,* and *S. mansoni* is relatively high, thus it should now be possible to identify markers that are universal among *Schistosoma* species (i.e., using DNA sequences conserved among species)*,* as well as other markers that are specific to species or species-groups (i.e., using DNA sequences that differ among species). Full genome-sequencing of additional species and specimens of *S. haematobium, S. japonicum,* and *S. mansoni* is desirable to better characterize differences within and among these species, to develop additional genetic markers, and to examine genes as well as conserved non-coding elements associated with drug resistance.

## Background

Schistosomiasis is caused by blood flukes of the genus *Schistosoma*, affecting about 210 million people inhabiting 76 countries with tropical and sub-tropical climates [1]. Urogenital schistosomiasis caused by *Schistosoma haematobium* affects 112 million people, with cases widely distributed across Africa [2]. Several efforts are underway to control *S. haematobium*, including the Schistosomiasis Consortium for Operational Research and Evaluation (SCORE) program (http://score.uga.edu/index.html), which was established in 2008 to answer strategic questions about schistosomiasis control and elimination. New tools are needed to monitor changes in population structure of the *Schistosoma* parasite subjected to various levels of drug pressure [3]. Molecular tools, in particular, will allow researchers to determine whether changes in gene frequencies provide insight into the effectiveness of treatment, understand the impacts of treatment on the gene pool and population structure of *Schistosoma* parasites, and establish whether movement of humans from refugia or non-treated areas introduces new parasites into local populations [4].

Currently, only a single drug, praziquantel, treats schistosomiasis [5]. Cure rates vary but rarely reach 100%, and some infections may persist even with frequent treatment [6]. One goal of the SCORE program is to understand and document whether changes in drug tolerance arise during different drug administration programs, and specific genetic assays are needed to better monitor population structure and potential changes in *Schistosoma* populations under praziquantel treatment.

Genetic resources exist for studying these aspects of *S. mansoni* and *S. japonicum,* due to the availability of the full genome sequence for each [7,8] and the relatively high level of research devoted to these species [4]. The recently published genome sequence for *S. haematobium*[9] was not available when we initiated this research, but even with this important new resource, relatively few validated genetic markers exist to quantify and analyze genetic variation of *S. haematobium* populations, creating a significant knowledge gap for this species. Because several large-scale SCORE projects focus on *S. haematobium*, obtaining larger-scale genetic and genomic resources enabling the study of *S. haematobium* at the population-level is desirable. Thus, we produced a large-scale database with a validated subset of DNA markers for *S. haematobium* to create the resources needed for future work by both the SCORE program and the larger research community.

Here, we focused on characterizing a type of highly variable DNA marker in widespread use for more than 20 years - microsatellite DNA loci [10] - which are the most actively used genetic marker within the schistosomiasis research community reviewed by Rollinson *et al*. [11]. Although 16 microsatellite loci have been developed for *S. haematobium* and seven have been used in large-scale studies (Gower *et al*. [3,12]), only three have proven robust in our hands. We sought to develop numerous microsatellite loci from throughout the *S. haematobium* genome for long-term use, and to identify a superior small subset of 10–20 loci for immediate, routine use. Thus, our general strategy was to collect DNA sequence data from relatively long-reads of random DNA fragments totaling about 1x genome coverage using a Roche 454 FLX Genome Sequencer and to identify microsatellite DNA markers from the sequences using two different techniques. We created a database containing thousands of potential microsatellite markers, and we validated a random selection of 32 markers from the database using DNA collected from 6 widely dispersed populations of *S. haematobium*. We show that the markers in the database can be used to obtain highly variable microsatellite DNA genotypes of *S. haematobium* and enable population-level analyses across the range of *S. haematobium* in Africa.

## Methods

### *Schistosoma haematobium* samples

#### Genome sequencing sample

Because it is best to use DNA derived from single genotypes for genome sequencing, 90 laboratory bred *Bulinus wrighti* snails were individually exposed to single *S. haematobium* miracidia collected from urine samples of school children in Zanzibar. A large number of snails were used because of the expected low proportion of successful infections from single miracidia and the possibility of snail death prior to sufficient clonal cercariae being collected. The infected snails were maintained in the laboratory for 33 days in standard conditions (water-filled trays at 27°C, 12 h/12 h light/dark), and then transferred to individual vessels and placed in the dark. The individual snails were placed in fresh snail water and brought into the light every 2–3 days to stimulate shedding of the cercariae. Eight snails were found shedding cercariae and over a period of 28 days, cercariae from each individual snail were collected using a pipette to capture them in a minimal water volume. Cercariae from each snail were collected in individual 1.5 ml microcentrifuge tubes, centrifuged and stored at -20°C after excess water was removed. Cercariae from each individual snail collected over multiple days were combined for the extraction procedure. The results presented here are based on DNA purified from 3703 cercariae (the total cercariae collected from a single snail over a 28 day period) using DNeasy blood and tissue kits (Qiagen, Valencia, CA). We eluted DNA from the DNeasy columns using two aliquots of 50 μL of Buffer AE, according to manufacturer instructions, and the DNA purification procedure yielded approximately 3.1 μg of DNA, measured with a Nanodrop ND1000 spectrophotometer.

#### Genetic survey samples

To identify polymorphic microsatellite DNA loci in the random subset and survey genetic variation among *S. haematobium* populations, adult worms from six African countries (Table 1) were obtained from the schistosomiasis collection at the Natural History Museum (SCAN) [13]. These isolates had been passaged through golden hamsters and *B. wrightii* in the Natural History Museum (NHM) laboratory and stored in liquid nitrogen. DNA was extracted individually from six adult male and six adult female worms from each of the six localities (Table 1) using the DNeasy blood and tissue kit (Qiagen, Inc.), as described above. To conserve the limited supply of DNA, we performed whole genome amplifications (WGA) of 1 μL (~10 ng) purified DNA using the REPLI-g UltraFast mini Kit (Qiagen, Crawley, UK), and we diluted the resulting WGA amplifications 1:24 prior to using diluted, WGA DNA as a template for microsatellite locus genotyping.

**Table 1:** **Sampling details for the six populations of *Schistosoma haematobium *** **studied**

**Population**	**Latitude/Longitude**	***ID***	**Year sampled**	***Source***
Senegal	16°37’00”N/15°01’60”W	NHM3292	1995	Human urine
Zanzibar	05°58’15”S/39°18’29”E	NHM3739	1999	Human urine
Malawi	14°13’60”S/33°33’00”E	NHM880	1998	Unknown
Mauritius	20°09’53”S/57°30’47”E	NHM2576	1992	Human urine
Nigeria	11°57’54”N/08°15’00”E	NHM682	1985	*Bulinus truncatus*
South Africa	27°00’00”S/32°50’00”E	NHM812	1986	Unknown

### 454 sequencing

To provide low coverage genome sequences of *S. haematobium* and to identify microsatellite loci, we converted the DNA extracted from the single, pooled sample of 3703 cercariae shed from an individual snail into a 454 sequencing library by ligating Roche 454 titanium blunt MID-tagged adaptors (RL kits were not yet available) to the DNA according to the manufacturer’s instructions. Due to limited amounts of genomic DNA, we reduced the stringency of the 454 size-selection conditions, prior to sequencing, to retain smaller fragments in the resulting sequencing library. We sequenced the size-selected library using titanium chemistry on a Roche 454 FLX Genome Sequencer according to the manufacturer’s instructions.

### Sequence filtering, assembly, and microsatellite identification

Following sequencing, we trimmed low quality sequence data using default parameters of the Roche v2.3 analysis pipeline, and we assembled trimmed reads to contigs using gsAssembler v.2.3 software. We used RepeatMasker (http://www.repeatmasker.org) and MsatCommander [14] to identify independently microsatellite loci within resulting contigs, and we merged results of the two searches to generate a general feature format (GFF) file containing the microsatellite information.

### Comparative mapping of *S. haematobium* contigs to the *S. mansoni* genome

Because the *S. haematobium* genome sequence was not publicly available at the time and because we wanted to (1) determine the proximity of microsatellite loci to annotated or putative genes, (2) select putatively unlinked microsatellite loci for testing, and (3) compare the frequency and size distribution of microsatellite motifs across species, we mapped the *S. haematobium* contigs to the *S. mansoni* genome using SHRiMP [15] and BWA [16]. This approach assumes significant synteny between the two species, which the mapping results suggest is true - an observation also reported by Young *et al*. [9]. We then designed primers adjacent to microsatellites having sufficient flanking sequence (> 20 bp) using Primer3 [17] and a modified version of MsatCommander (http://github.com/faircloth-lab/msatcommander-gs), which designs tagged primers for polymorphism testing using a customized Primer3 workflow. We converted this information to the GFF format and put all the information together in a single GFF file.

We selected a random subset of 32 tagged primers from the database (Additional file 1: Table S1) for polymorphism testing by choosing primers designed from the subset of contigs containing only one microsatellite locus and mapping onto the *S. mansoni* genome (using BWA) with a map quality score of ≥ 30.

### Testing polymorphism and surveying genetic variation in *S. haematobium*

We screened 32 primer pairs for amplification and polymorphism against DNA from eight of the 72 *S. haematobium* specimens. We performed PCR amplification in 12.5 μL volume reactions with 10 mM Tris pH 8.4, 50 mM KCl, 25.0 μg/ml BSA, 0.36 μM unlabeled primer, 0.04 μM tag labeled primer, 0.36 μM universal dye-labeled primer, 3.0 mM MgCl_2_, 0.8 mM dNTPs, 0.5 units JumpStart Taq DNA Polymerase (Sigma), and ~20 ng WGA DNA template using an Applied Biosystems GeneAmp 9700. We amplified all loci using one of three touchdown PCR protocols [18], TD65, TD60, or TD55 (Table 2). Each touchdown protocol included a 10°C span of annealing temperatures (65-55°C, 60°C-50°C, and 55-45°C respectively), and touchdown cycling parameters consisted of 20 cycles of 95°C for 30s, highest annealing temperature (decreased by 0.5°C per cycle) for 30s, and 72°C for 30s; and 20 cycles of 95°C for 30s, lowest annealing temperature for 30s, and 72°C for 30s. We ran all PCR products on an ABI-3730xl sequencer with Naurox size standard prepared as described in DeWoody *et al*. [19] except that all Naurox reverse primers started with GTTT. We used Genemapper version 4.0 (Applied Biosystems) to analyze the results and determine genotypes.

**Table 2:** **Details for 15 polymorphic microsatellite loci developed for *Schistosoma haematobium***

**Locus**	**Primer Sequence 5′ -- > 3′**	**Repeat motif**	**T**_**A**_	**Size (bp)**	**N**	***k***
Shae_01	F: †GCATCCAATTTCGTACAC	AAT	TD60	257 - 307	65	12
	R: *CCACATTAGGCCAACAAG					
Shae_02	F: *TTAGTGTGTTTGGCTTCAAC	AAT	TD60	183 – 225	69	9
	R: †CCTCGAATGAAATCCTGAC					
Shae_03	F: †GCTGAGCTTGAGATTG	AAT	TD55	291 – 334	62	15
	R: *CTTCTGTCCCATCGATACC					
Shae_04	F: †CCCATCGCTGATATTAAAG	AAT	TD65	289 – 325	70	10
	R: *TCTAGTCGTCTTGGGATCC					
Shae_05	F: *TGTGCACAAGAAAGATTAAATG	AAT	TD65	288 – 319	68	10
	R: †ACGACAATGTTGCAAGTTC					
Shae_06	F: †GGTGGATTACGCAATAG	AC	TD65	333 – 347	67	8
	R: *TTTAATCAACCGGGTGTC					
Shae_07	F: *TCCAAGCACCATTATCAAG	AAT	TD65	315 – 330	66	6
	R: †ACGGAAACTTGTTGAAATG					
Shae_08	F: *CTAAACTGGCAAGATTTC	AAT	TD65	299 – 330	68	11
	R: †CAACGTGCCTTTATTTC					
Shae_09	F: †GGGATGTATGCAGACTTG	AAT	TD65	213 – 256	68	13
	R: *TTGTTTGGCTGCAGTAAC					
Shae_10	F: †CGCATGTCATACCTATCTCC	AAT	TD65	198 – 213	69	6
	R: *GCTTATCAGGCCTATCTCC					
Shae_11	F: *TTGGTTTAGAAATTACATCACC	ATC	TD65	207 – 222	66	5
	R: †CCAACAATATTAATGGACAGC					
Shae_12	F: †CGTCTTAGTGAGCCAGATG	AAC	TD65	257 – 278	68	6
	R: *CTCGTGGACATCATCAG					
Shae_13	F: *GAGCAGCTATTTCGTATCG	AAT	TD60	183 – 225	69	12
	R: †ACCGTGGACAGTTCATCAG					
Shae_14	F: *GTCCTCCTTCCCTCTTTG	ACTC	TD65	210 – 254	67	10
	R: †CACATTCGTCCTAGATATCG					
Shae_15	F: *CTTTCAGTAGGATTTGTTG	ATC	TD65	300 – 322	67	9
	R: †CGACGTCAAGCACTGTAC					

* indicates CAG tag (5′- CAGTCGGGCGTCATCA-3′).

† indicates pigtail (5′- GTTT-3′).

The annealing temperature (**T**_**A**_) where TD65, TD60, and TD55 indicates touchdown protocols with a highest annealing temperature of 65°C, 60°C, and 55°C, respectively; **size** indicates the range of sequenced alleles in base pairs plus the length of the CAG tag; the number of individuals genotyped out of 72 is **N**; ***k*** is the number of alleles observed.

Fifteen primer pairs (47%) amplified high quality, unambiguous, PCR products and displayed polymorphisms. Using these 15 loci, we genotyped 72 individuals (12 individuals sampled from 6 locations) using the reaction conditions described above with WGA DNA (Table 2). To test for potentially negative effects of using WGA DNA, we also screened genomic DNA from 24 individuals at 3 loci (Shae_03, Shae_13, and Shae_15). We did not detect differences between genotypes derived from genomic DNA or WGA DNA.

### Analysis of Microsatellite diversity within and among populations

To measure the degree of genetic polymorphism within populations, we calculated expected heterozygosity (H_E_), observed heterozygosity (H_O_), and mean number of alleles per locus (MNA) using MICROSATELLITE TOOLKIT [20]. We tested for deviations from Hardy-Weinberg equilibrium (HWE) in H_E_ and H_O_ using tests of heterozygote deficiency (U-test) at an α of 0.05 in GENEPOP web version 4.0.10 [21,22]. To estimate the degree of inbreeding within populations, we calculated mean *F*_IS _[23] for each population in GENEPOP, and we used GenAlEx 6 [24] to calculate the number of private alleles within each population.

Although many population differentiation statistics have been proposed for use with microsatellites, we chose to calculate and compare two measurements of genetic differentiation: F_ST_ and D_est_. *F*_ST_ is relatively common in the literature but is often criticized because of its tendency to underestimate genetic differentiation when markers are highly variable, which is often the case with microsatellites [25,26]. *R*_ST_[27] is also commonly reported in studies that use microsatellite markers because it is not affected by the amount of genetic variability within populations. However, *R*_ST_ does not perform well when sample sizes are small [28], and we opted not to calculate this parameter in our study. In place of *R*_ST,_ we chose to calculate D_est_[29], which does not underestimate differentiation when markers are highly variable [29]. We used GenAlEx 6 to calculate pairwise *F*_ST_ using analysis of molecular variance (AMOVA) with 999 permutations, and we used SMOGD [30] to calculate pairwise, harmonic mean D_est_ values. To test for a pattern of isolation by distance (IBD), we used Isolde in GENEPOP to examine the correlation between Slatkin’s linearized *F*_ST_ [*F*_ST_ /(1- *F*_ST_)] [27] and the respective geographic distance between sampling locations.

We used GenAlEx 6 to determine the probability of identity (PI), which describes how well a locus can distinguish among genetically different individuals (a low PI indicates powerful genetic markers). To identify loci with potential null alleles and estimate null allele frequencies we used MICROCHECKER v2.2.3 [31] in combination with the Brookfield estimator [32], which assumes that there are no homozygote null alleles and that the absence of a genotype is caused by degraded DNA, genotyping error, etc. To determine which loci provided the most information about population differentiation, we used a log-likelihood ratio (*G*) based exact test in GENEPOP, and we compared the statistical significance (α = 0.001) of pairwise *F*_ST_ values for each locus to the respective statistical significance of pairwise *F*_ST_ values across all loci. Because all across-loci pairwise *F*_ST_ values were statistically significant, we counted the number of populations with pairwise *F*_ST_*P* > 0.001 (α = 0.001) to measure disagreement between locus-specific estimates of population differentiation and population-specific estimates of differentiation. Across all populations we calculated mean allelic richness using MICROSATELLITE ANALYZER (MSA) v.4.05 [33].

To provide a relative rank of the utility of each locus to all other loci, we selected several desirable characteristics for ranking, which we weighted equally: low *PI*, low number of populations having null alleles, low number of disagreements with statistical significance of population structure, and high mean number of alleles. We then scored all loci, by ranking each locus within all categories from most desirable (1) to least desirable (15). We added rank values together to create a final score for each locus, and we ordered loci from lowest values of this final score (most informative) to highest (least informative).

## Results and discussion

### 454 sequences

We obtained 1,058,114 reads totaling 299,226,826 bases from 454 sequencing. Average read length was 283 bases, which is lower than usual for titanium sequencing but is expected due to our reduction of size-selection stringency during library preparation. The *S. haematobium* genome is about 385 megabases (Mb; [9]), which is between *S. mansoni* at 363 Mb [7] and *S. japonicum* at 397 Mb [8]. Thus, the 454 sequences we obtained represent about 0.78x coverage. This low-level of coverage is ideal for discovering a large proportion of unique loci (i.e., each read has a high probability of sampling part of the genome for the first time), but is not, on its own, intended for genome assembly because only about half of the genome will be included in the sequences obtained. Even at this low coverage, some DNA fragments will be sequenced multiple times, especially repetitive elements. After read filtering, sequence assembly yielded 11,166 contigs >500 bases in length with an average size of 682 bases, an N50 of 642 bases, a maximum contig size of 5,900 bases, and 89% of bases ≥ Q40.

### Comparison of microsatellites among Schistosoma genomes

**Figure 1:**
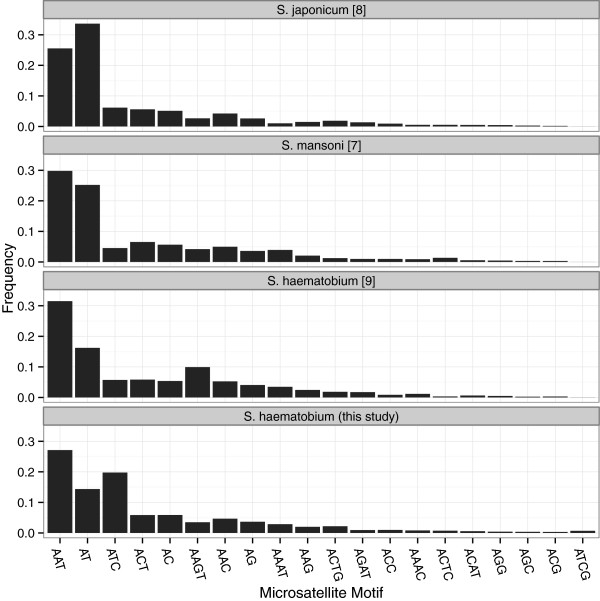
**Locations of *Schistosoma haematobium *population samples.** Vertical bars represent allelic richness, and horizontal bars represent heterozygosity (see Table 1 for location details).

We determined the frequency distribution of microsatellites by motif and repeat lengths for all microsatellites found in the *S. haematobium* 454 reads (this study) and the *S. haematobium* genome (obtained using Illumina sequencing [9]). In addition, we examined all di-, tri-, and tetra-nucleotide repeats in detail and compared them with *S. japonicum* and *S. mansoni* (Figure 1; Additional file 2: Table S2). *Schistosoma* species differed markedly in the frequency of AT repeats with AT repeats being the most common in *S. japonicum*, second most common in *S. mansoni*, and second or third most common in *S. haematobium*, depending on which data (454 or Illumina) are used. In comparison to the published *S. haematobium* genome (from a strain in long term culture originally isolated in Egypt and sequenced on the Illumina platform [9]), our sequences (from an individual deriving from Zanzibar and sequenced on the 454 platform), there are markedly more ATC repeats and fewer AAGT repeats in the 454 sequences. The frequency of AAGT repeats found in the 454 sequences is more similar to the frequencies found in *S. mansoni* and *S. japonicum* genomes. Thus, it is possible that the current full genome assembly of *S. haematobium* overestimates AAGT frequency. In contrast, the 454 sequences show far more ATC repeats than any of the full genome assemblies. Thus, the ATC repeats are either systematically overestimated in the 454 reads or underestimated in the Illumina reads used for *S. haematobium* and potentially all three *Schistosoma* genome assemblies produced so far.

SchistoDB [34]; http://SchistoDB.net provides a rich resource for comparisons among the three schistosomes with genome sequences available. Our sequences add to the possible comparisons, but our results illustrate that some caution should be used in these comparisons. There are many differences among sequencing platforms. Some of these differences are known from the chemistry and technologies employed within the instruments, but it is often difficult to estimate how these will impact specific research projects [35]. Multiple studies have, therefore, been conducted to directly compare sequencing results from multiple platforms e.g., [36-38]. Sequencing of additional specimens and sequencing with other chemistries and platforms is needed to determine whether the differences we observed are artifacts of the chemistry and platforms used or reflect biological differences among or within species.

### Microsatellite primers and database

We used two different approaches to identify microsatellites and design primers for those loci. In the first approach, the programs RepeatMasker and MsatCommander identified 18,235 and 17,739 microsatellites, respectively. SHRiMP mapping resulted in 144,450 reads mapped from the *S. haematobium* data to the *S. mansoni* genome. For each microsatellite within the mapped reads, we extracted 500 bp upstream and downstream of its location from the *S. mansoni* genome to identify primers adjacent to each locus. Primer3 yielded 13,790 pairs of primers for these loci.

In the second approach, microsatellite loci were identified and primers were designed from the *S. haematobium* 454 sequences using a modified version of MsatCommander that is tuned to genome-scale datasets. A total of 113,757 microsatellite loci were identified but collapsed into 84,754 loci that were >50 bp apart. Of those, 35,691 had >20 bp of preceding and trailing sequence (i.e., enough for potential primer design). The 454 reads were then mapped onto the *S. mansoni* genome (v 3.1), resulting in 114,015 reads with a BWA mapQ score ≥30. We then filtered those reads to include only those that had one matching location in the *S. mansoni* genome, contained a microsatellite, and had >20 bases of flanking sequence on each side of the repeat, resulting in 3,656 reads. From those, 3,148 primer pairs were designed, of which 1,479 were compatible with 5’ CAG-tags (Table 2). We further examined the map locations in *S. mansoni* to retain only one primer pair per scaffold, resulting in 403 primer pairs (Additional file 3: Table S3). We then randomized the list of 403 primer pairs and empirically tested the first 32.

We compiled results from analyses above into lists and searchable databases for distribution. Primers for microsatellite loci, their location within the genome and a BLAST [Alschul *et al.* 1997] server are available at: http://www.cebio.org/projetos/schistosoma-haematobium-genome.

The different approaches and lists of primer pairs may be useful for a variety of purposes. For example, the first approach demonstrates that large numbers of microsatellite repeats can be obtained using commonly available bioinformatics tools and that many of the *S. haematobium* reads can be mapped directly to the *S. mansoni* genome. Because the *S. haematobium* genome is more similar to the *S. mansoni* than *S. japonicum* genome [9,39], the 13,790 loci identified from the SHRiMP alignment represent a large potential pool of markers that could be mapped to *S. mansoni*, which is 4 times larger than the 3,148 primer pairs that were mapped to *S. mansoni* from strategy 2. The major advantage of strategy 2, however, is that 1,479 primer pairs could easily be used with CAG-tags for low-cost, 3-primer PCR [40], and 403 of those could be used for linkage mapping in *S. haematobium* or *S. mansoni*. Some may find it curious why linkage mapping would be necessary when a genome sequence is available, but the *Schistosoma* genomes are not yet finished (i.e., not all sequences are assembled into chromosomes) and finishing generally relies upon data that are independent of the sequences to validate the computational assembly of the short DNA sequence reads. Finally, these primers provide potential markers for diagnosis, epidemiology and phylogenetic analysis of Schistosoma species cf., [41-43].

### Population genetic survey

#### DNA sample quality

Fifteen of the 32 primer pairs we screened yielded high-quality polymorphic markers with the initial set of 8 DNA samples. We used these 15 polymorphic markers to screen DNA from 72 *S. haematobium* individuals (six male worms and six female worms each from six locations; Table 1, Table 2, Additional file 4: Table S4). Three worms appeared to have low-quality DNA or contained significant amounts of PCR inhibitors (we obtained genotypes from zero, four, and four loci in each these three individuals). All other samples yielded data from at least 10 loci (one individual each producing amplicons at 10, 11 and 12 loci, all others having genotypes from 14 or all 15 loci). We concluded that the three DNA samples that yielded few or no genotypes were of low quality, and we excluded them from further analysis. We retained the three samples scored at 10–12 loci because it is possible the failure of loci to amplify in these samples results from the presence of null alleles. However, it is also possible that the failure of loci to amplify in these samples results only from their lower quality, potentially inflating null allele estimates. Thus, primer pairs with reduced numbers of individuals scored (Table 2) may reflect a higher frequency of null alleles or reduced robustness of the PCR conditions to amplify lower-quality DNA.

WGA of *Schistosoma* DNA has been demonstrated to provide reliable templates for genotyping microsatellite loci [44]. WGA is useful because it increases the small amount of DNA obtained from individual worms, eggs, cercariae or miracidia to quantities that allow multiple PCRs, replicate PCRs and thus estimation of genotyping error rates [44]. However, in the current study, while using WGA DNA resulted in more template for PCRs, and thus more PCRs per sample, it did not increase the number of usable genotypes. In fact, we had complete congruence in genotypes called from WGA and genomic DNA suggesting WGA was unnecessary, and only helped us in confirming that the genotypes were repeatable. It is possible that inhibitors were carried through WGA for the 6 samples that yielded relatively few genotypes, but it is also possible that the starting DNA was not sufficient in these samples and yielded amplification of only part of the genome instead of the whole genome. In the future, if there is concern that genomic DNA is of low quality and may result in null alleles and poor amplification we would recommend initially trying WGA on a small subset of samples to see if amplification is improved. Further work on the specific conditions where WGA will improve genotyping for various *Schistosoma* samples and species is needed.

#### Genetic variability of *Schistosoma haematobium*

We analyzed genotypes of 69 individuals across the six locations sampled (Table 1). The total number of alleles per locus ranged from 5 for Shae_11 to 15 for Shae_03 (Table 2). The largest number of alleles was in the Zanzibar population (average = 5.7, Table 3), whereas the lowest number of alleles occurred in the South African population (average = 1.3, Table 3). South Africa was also an outlier for expected heterozygosity (6%, Table 3); all other populations had an *H*_E_ between 51% and 70%. We did not find differences between expected and observed heterozygosities (*P* = 0.356), and no populations showed evidence of departures from HWE. We identified private alleles in all six populations (Table 3), occurring at a minimum frequency of 0.10. Four populations had slightly negative *F*_IS_ values whereas two had slightly positive *F*_IS_ values (Table 3), but none were significantly different from zero. The limited sample sizes used here limit the power to detect deviations from HWE, but the lack of trends in one direction or the other suggest that neither the markers nor samples were systematically biased.

**Table 3:** **Summary of genetic variation at 15 microsatellite loci in 6 populations of *Schistosoma haematobium***

**Population**	***N***	***H***_**E**_	***H***_**O**_	***A***	***P***	***F***_**IS**_
Senegal	12	0.51 ±0.06	0.51 ±0.04	3.3	7	-0.01
Zanzibar	12	0.70 ±0.04	0.63 ±0.04	5.7	18	0.10
Malawi	10	0.60 ±0.07	0.65 ±0.04	3.9	8	-0.08
Mauritius	11	0.62 ±0.04	0.65 ±0.04	4.1	6	-0.06
Nigeria	12	0.58 ±0.06	0.58 ±0.04	4.4	7	0.01
South Africa	12	0.06 ±0.04	0.06 ±0.02	1.3	3	-0.02

*N* is the sample size (number of adult *S. haematobium* genotyped, DNA from 6 males and 6 females was sampled), *H*_E_ is the expected heterozygosity (calculated as Nei’s unbiased gene diversity (Nei, 1987)) ± the inter-locus standard deviation, H_O_ is the observed heterozygosity ± the interlocus standard deviation, *A* is the mean number of alleles, *P* is the number of private alleles, and *F*_IS_ is the Weir and Cockerham (1984) estimate for inbreeding.

The variability that we find with these 15 polymorphic loci is likely to be as high or higher than that found with loci developed previously e.g., [3,45]. It is important to note that our sampling is broader and shallower than previous studies of microsatellite loci in *S. haematobium *[3,45]. Direct comparisons of these two primer sets genotyped in the same samples will be needed to determine definitively which are more powerful. Of critical importance, however, is that the loci we describe are not just variable, but the loci are also easy to amplify and score (see below). We are now routinely using multiplexes of these 15 loci to genotype *S. haematobium* for a variety of ongoing studies and all 15 loci are proving robust for all *S. haematobium* life cycle stages and are easily scored. Thus, the primers identified in Table 2 provide a significant new resource for researchers of *S. haematobium*.

#### Population structure of *Schistosoma haematobium*

Pairwise *F*_ST_ values suggest strong population structure (Table 4). The most differentiated populations were Senegal and South Africa (*F*_ST_ = 0.65, *P* = 0.001), whereas the least differentiated populations were Zanzibar and Mauritius (*F*_ST_ = 0.16, *P* = 0.001). All pairwise *F*_ST_ values were statistically significant at an alpha of 0.001. We did not detect a pattern of isolation by distance (R^2^ = 0.01, *P* = 0.35). Pairwise harmonic mean *D*_est_ values suggested strong population structure and exhibited a pattern of differentiation similar to pairwise *F*_ST_ values, suggesting South Africa and Senegal as the most differentiated populations (*D*_est_ = 0.73) and Zanzibar and Mauritius as the least differentiated populations (*D*_est_ = 0.18). P-values are not available for estimates of *D*_est_, but their similarity to *F*_ST_ and large value suggests that *S. haematobium* from all of the sampled localities should be viewed as significantly different.

**Table 4:** **Pairwise differentiation among populations of *Schistosoma haematobium***

**Populations**	**Senegal**	**Zanzibar**	**Malawi**	**Mauritius**	**Nigeria**	**South Africa**
Senegal		0.41	0.64	0.35	0.28	0.73
Zanzibar	0.27		0.25	0.18	0.33	0.53
Malawi	0.36	0.17		0.32	0.44	0.33
Mauritius	0.29	0.16	0.22		0.30	0.51
Nigeria	0.26	0.19	0.22	0.20		0.44
South Africa	0.65	0.50	0.46	0.54	0.49	

*F*_ST_ values are below diagonal (all P-values ≤ 0.001) and pairwise harmonic mean *D*_est_ values are above diagonal.

These results are consistent with previous findings [3,34,35,45]. Gower *et al. *[45] found an average *F*_ST_ of 0.173 among the five countries they investigated (Cameroon, Kenya, Mali, Niger, and Tanzania). Our *F*_ST_ values are higher than those found by Gower *et al. *[45], but it is important to note that the markers (loci) used and sampling intensity heavily influence resulting measures of differentiation [26]. Additionally, highly polymorphic microsatellite loci constrain *F*_ST _[26], which makes measures such as *D*_est_ more appropriate [29]. Despite these constraints, *F*_ST_ and *D*_est_ values are quite similar in this study (Table 4). Indeed, we would draw the same conclusions from either measure – there is very high differentiation among all populations, but differentiation is especially high between South Africa and all other populations. We would also draw the same conclusion of significant population differentiation among *S. haematobium* populations in different African countries from the results of Gower *et al. *[45] or our study.

#### Ranking loci by information content

Although all of the loci we tested are useful, some researchers may choose to use only a subset of these 15 loci. Thus, we sought to compute relative utility of each locus. We measured four parameters across the 15 microsatellite loci (Table 5). The *PI* ranged from 0.21 for Shae_14 to 0.61 for Shae_04. Five loci exhibited evidence of null alleles in one or two populations. In populations that demonstrated evidence of null alleles, null allele frequency ranged from 0.16 (Shae_09, Mauritius) to 0.33 (Shae_10, Senegal). No single locus estimated significant differentiation across all population pairs. The number of population pairs with an *F*_ST_*P*-value greater than 0.001 ranged from 3 (Shae_15) to 10 (Shae_11 and Shae_13). The mean number of alleles across populations ranged from 3.52 (Shae_11) to 8.24 (Shae_08). Our usefulness scores ranged from 8 (Shae_08, most useful) to 36 (Shae_11, least useful) allowing reasonable discrimination among loci. Thus, if researchers wanted to score a subset of these loci, they now have a simple way to prioritize which loci to include.

**Table 5:** **Summary statistics of 15 microsatellite loci screened in 6 populations of *Schistosoma haematobium***

**Locus**	***PI***	**Populations with null alleles**	**Pairwise *F***_**ST **_**with *p*** **> 0.001**	***A***	**Score**	**Rank of usefulness**
Shae_01	0.22	0	4	6.9	9	2
Shae_02	0.37	2	7	6.4	23	8
Shae_03	0.30	0	6	7.6	12	3
Shae_04	0.25	0	4	7.0	9	2
Shae_05	0.36	0	3	6.8	15	6
Shae_06	0.42	0	4	5.6	22	7
Shae_07	0.44	1	8	4.5	32	12
Shae_08	0.28	0	4	8.2	8	1
Shae_09	0.34	2	5	6.8	16	5
Shae_10	0.36	2	7	4.7	27	10
Shae_11	0.49	0	10	3.5	36	14
Shae_12	0.61	1	7	4.5	35	13
Shae_13	0.43	0	10	5.4	29	11
Shae_14	0.21	0	5	6.3	13	4
Shae_15	0.59	0	3	5.4	27	9

*PI* is the probability of identity, the number of populations with expected null alleles present, the number of populations with non-significant (*p* > 0.001)F_ST_ values (significance determined by G-based exact test), *A* is the mean number of alleles, Score is determined from rank-sum of desirable characteristics (e.g., allelic diversity, lack of predicted null alleles, etc.; see text for details), Rank of Usefulness is the rank order for loci based on Score (lowest Score and rank is best), loci with the same rank indicate loci with identical rank sums.

#### Comparison to other studies and primers

We have developed microsatellite loci from literally hundreds of species using a variety of approaches during the past 20 years [46-48]. It is common for about half of the primer pairs we tested to yield amplifiable, easily genotyped, and polymorphic loci. It is not unusual, however, for the proportion to be substantially lower than 50%, but it is somewhat uncommon for the proportion to be substantially higher than 50%. Here, we focused on single-copy loci which are likely to have been sequenced only a single time. Because 454 sequences have an average error rate of about 1% per base, this means that about 18% (1–0.99^20^) of 20-base primer sequences will contain at least one error. Errors in primer sequences will be enhanced when they are at the ends of a sequence read (where quality is lowest), but the low-quality bases are mostly mitigated by matching the low-coverage *S. haematobium* 454 sequences to high-coverage *S. mansoni* genomic data. Indeed, most primers we design typically amplify (~75%), but it is well-known that the proportion of variable loci varies greatly among taxa and that mutation rates vary among loci and is correlated with allele length [49]. We focused on tetranucleotide repeats because they are generally easy to genotype [50]. Tetranucleotide repeats also have the advantage of having the lowest mutation rate [51] and thus the lowest level of homoplasy.

We developed a customized pipeline to design primers and identify primer pairs from regions of interest. This pipeline builds on the easily implemented MSATCOMMANDER [14] program, but includes customization to help speed searches on genome-scale data and maintains the output in an SQL database. We did not attempt multiple PCR conditions that could optimize the amplification of each locus. Instead, we focused on applying a small number of broadly useful PCR conditions on a moderate number of primer pairs. This strategy has the advantage of quickly focusing on primers that are likely to work well in multiplexes. It also provides a realistic assessment of the proportion of loci that are likely to work under normal lab conditions, rather than the largest possible proportion that could ever be made to work.

We have only tested 32 of the many thousands of primer pairs in our databases. If researchers need additional loci to address health-care challenges posed by urogenital schistosomiasis [52], we recommend investigating additional primers in the list of 403 loci mapped to *S. mansoni* scaffolds (Additional file 3: Table S3 *cf*. Additional file 1: Table S1) as the most productive avenue.

## Conclusion

**Figure 2:**
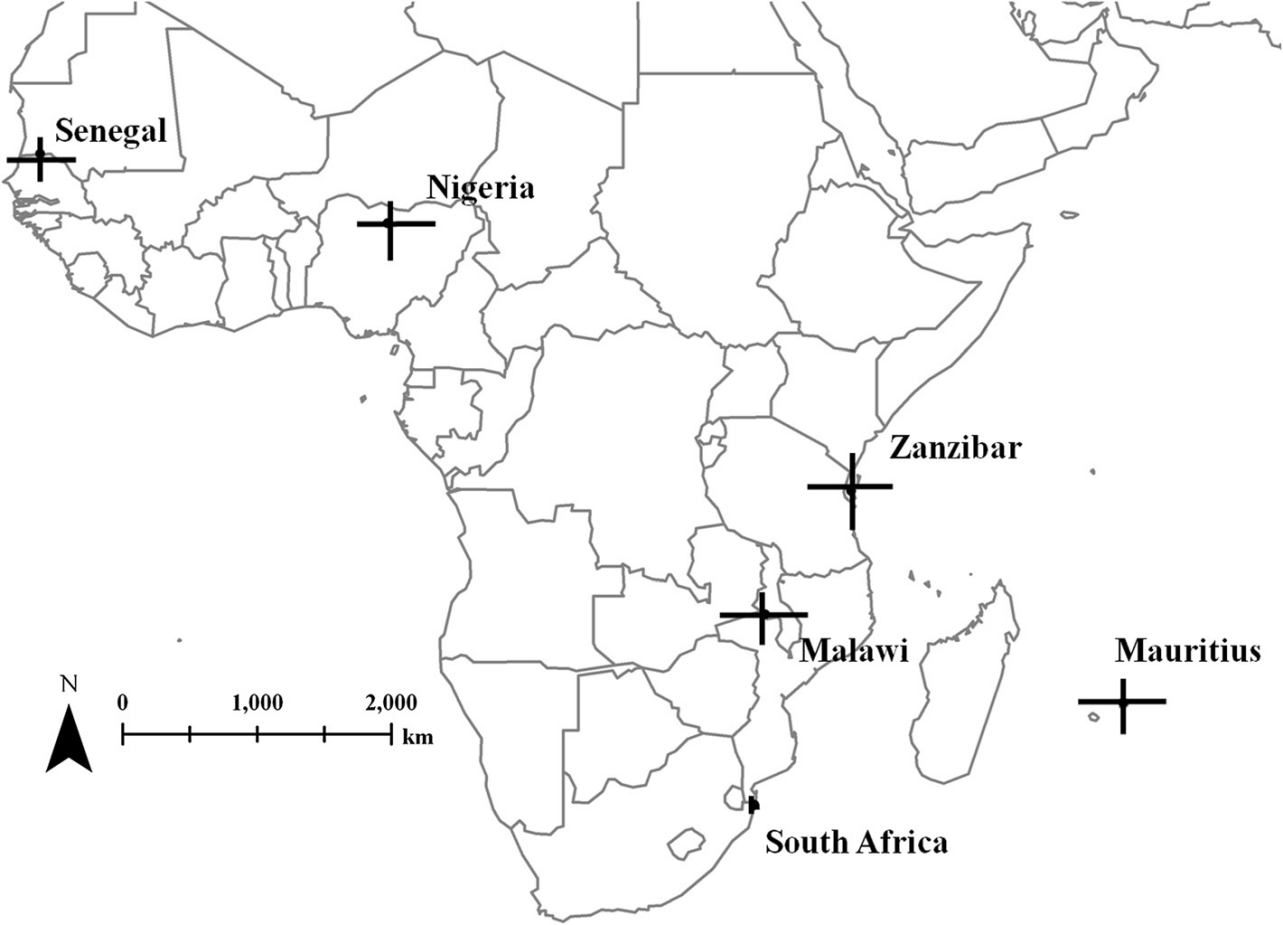
**Microsatellite motifs of *Schistosoma haematobium*.** Distribution of Repeat Types. Pattern of genetic diversity as measured with newly developed microsatellite DNA markers.

The highly variable DNA markers we have developed can be used to investigate the genetic epidemiology of *S. haematobium* within both definitive mammalian (worms, eggs and miracidia) and intermediate snail hosts (cercariae) from widespread geographical locations. These markers demonstrate that *S. haematobium* from five of the sampling locations are genetically diverse, whereas *S. haematobium* from South Africa has much lower diversity (Figure 2). *Schistosoma haematobium* from Zanzibar had the highest levels of genetic diversity, complementing the differences in mtDNA diversity found within and between *S. haematobium* populations from across mainland Africa and the Indian Ocean Islands [53,54]. An elimination programme is now ongoing in Zanzibar, and it will be interesting to see how the strong selection pressures imposed on parasite populations by praziquantel treatments impacts this diversity [55]. The sweep of markers in the panel of loci identified provides a novel microsatellite panel with the potential to be used to identify and track genetic determinants of drug resistance in *S. haematobium*. Thus, these markers are a valuable resource to SCORE and the wider community of researchers and healthcare professionals researching with an ultimate goal to eliminate urogenital schistosomiasis. Primers for microsatellite loci, their location within the genome and a BLAST server are available at: http://www.cebio.org/projetos/schistosoma-haematobium-genome.

## Competing interests

The authors declare no competing interests.

## Authors’ contributions

TCG, DR, SLL, GO and BCF designed the study; DR, BLW and AE conducted all sampling, husbandry, and DNA extractions; TCG and GGF staff conducted 454 sequencing; BCF designed primers tested; AZN and BCF conducted additional bioinformatics analyses; SLL genotyped all samples and AMM conducted microsatellite survey analyses. All authors wrote, read and approved the final manuscript.

## Authors’ information

TCG, SLL, AMM and BCF use and develop a variety of DNA tools, and have collaborated on a variety of microsatellite-based population genetic projects. DR, AE and BLW use molecular tools to study the biology and control of schistosomiasis. AZ and GO use bioinformatics tools and develop public databases to combat schistosomiasis.

## Supplementary Material

Additional file 1: Table S1 All 32 primer pairs screened for amplification, variation, and scorability are summarized in this excel workbook. Click here for file

Additional file 2: Table S2 Counts of microsatellite motifs found in three species of *Schistosoma* (*S. haematobium, S. japonicum,* and *S. mansoni*) are summarized in this excel workbook. Click here for file

Additional file 3: Table S3 All 403 primer pairs for *S. haematobium* that match to a single *S. mansoni* scaffold. Click here for file

Additional file 4: Table S4 Genotypes of the 72 *S. haematobium* samples used in this study, the reduced set of 69 analyzed, and output from population differentiation measures obtained from GenAlEx are given in this excel workbook. Click here for file

## References

[B1] Steinmann P Keiser J Bos R Tanner M Utzinger J Schistosomiasis and water resources development: systematic review, meta-analysis, and estimates of people at risk Lancet Infect Dis 2006 6 411 425 10.1016/S1473-3099(06)70521-7 16790382

[B2] van der Werf MJ De Vlas SJ Brooker S Looman CWN Nagelkerke NJD Habbema JDF Engels D Quantification of clinical morbidity associated with schistosome infection in sub-Saharan Africa Acta Trop 2003 86 125 139 10.1016/S0001-706X(03)00029-9 12745133

[B3] Gower C Gabrielli A Sacko M Dembelé R Golan R Emery A Rollinson D Webster J Population genetics of *Schistosoma haematobium*: development of novel microsatellite markers and their application to schistosomiasis control in Mali Parasitology 2011 138 978 994 10.1017/S0031182011000722 21679489

[B4] Rollinson D A wake up call for urinary schistosomiasis: reconciling research effort with public health importance Parasitology 2009 136 1593 10.1017/S0031182009990552 19627633

[B5] Fenwick A Rollinson D Southgate V Implementation of human schistosomiasis control: challenges and prospects Adv Parasitol 2006 61 567 622 16735173 10.1016/S0065-308X(05)61013-5

[B6] Doenhoff M Hagan P Cioli D Southgate V Pica-Mattoccia L Botros S Coles G Tchuem Tchuenté LA Mbaye A Engels D Praziquantel: its use in control of schistosomiasis in sub-Saharan Africa and current research needs Parasitology 2009 136 1825 1835 10.1017/S0031182009000493 19281637

[B7] Berriman M Haas BJ LoVerde PT Wilson RA Dillon GP Cerqueira GC Mashiyama ST Al-Lazikani B Andrade LF Ashton PD Aslett MA Bartholomeu DC Blandin G Caffrey CR Coghlan A Coulson R Day TA Delcher A DeMarco R Djikeng A Eyre T Gamble JA Ghedin E Gu Y Hertz-Fowler C Hirai H Hirai Y Houston R Ivens A Johnston DA The genome of the blood fluke *Schistosoma mansoni* Nature 2009 460 352 358 10.1038/nature08160 19606141 PMC2756445

[B8] Zhou Y Zheng H Chen Y Zhang L Wang K Guo J Huang Z Zhang B Huang W Jin K Dou T Hasegawa M Wang L Zhang Y Zhou J Tao L Cao Z Li Y Vinar T Brejova B Brown D Li M Miller D Blair D Zhong Y Liu F Hu W Wang Z Song H Chen S The *Schistosoma japonicum* genome reveals features of host–parasite interplay Nature 2009 460 345 351 10.1038/nature08140 19606140 PMC3747554

[B9] Young ND Jex AR Li B Liu S Yang L Xiong Z Li Y Cantacessi C Hall RS Xu X Whole-genome sequence of *Schistosoma haematobium* Nature Genet 2012 44 221 225 10.1038/ng.1065 22246508

[B10] Tautz D Hypervariability of simple sequences as a general source for polymorphic DNA markers Nucl Acids Res 1989 17 6463 6471 10.1093/nar/17.16.6463 2780284 PMC318341

[B11] Rollinson D Webster JP Webster B Nyakaana S Jorgensen A Stothard JR Genetic diversity of schistosomes and snails: implications for control Parasitology 2009 136 1801 1811 10.1017/S0031182009990412 19631013

[B12] Gower C Gabrielli A Sacko M Dembelé R Golan R Emery A Rollinson D Webster J Population genetics of *Schistosoma haematobium*: development of novel microsatellite markers and their application to schistosomiasis control in Mali – CORRIGENDUM Parasitology 2012 139 962 10.1017/S0031182011000722 21679489

[B13] Emery AM Allan FE Rabone ME Rollinson D Schistosomiasis collection at NHM (SCAN) Parasit Vectors 2012 5 185 10.1186/1756-3305-5-185 22943137 PMC3453491

[B14] Faircloth BC MSATCOMMANDER: detection of microsatellite repeat arrays and automated, locus-specific primer design Mol Ecol Resour 2008 8 92 94 10.1111/j.1471-8286.2007.01884.x 21585724

[B15] Rumble SM Lacroute P Dalca AV Fiume M Sidow A Brudno M SHRiMP: accurate mapping of short color-space reads PLoS Comp Biol 2009 5 e1000386 10.1371/journal.pcbi.1000386 PMC2678294 19461883

[B16] Li H Durbin R Fast and accurate short read alignment with Burrows–Wheeler transform Bioinformatics 2009 25 1754 1760 10.1093/bioinformatics/btp324 19451168 PMC2705234

[B17] Rozen S Skaletsky H Primer3 on the WWW for general users and for biologist programmers Meth Mol Biol 2000 132 365 386 10.1385/1-59259-192-2:365 10547847

[B18] Don R Cox P Wainwright B Baker K Mattick J 'Touchdown’PCR to circumvent spurious priming during gene amplification Nucl Acids Res 1991 19 4008 10.1093/nar/19.14.4008 1861999 PMC328507

[B19] DeWoody JA Schupp J Kenefic L Busch J Murfitt L Keim P Universal method for producing ROX-labeled size standards suitable for automated genotyping Biotechniques 2004 37 348 352 15470886 10.2144/04373BM02

[B20] Park SDE The Excel Microsatellite Toolkit Trypanotolerance in West African Cattle and the Population Genetic Effects of Selection 2001 University of Dublin, Dublin, Ireland: Ph.D. Thesis

[B21] Raymond M Rousset F GENEPOP (version 1.2): population genetics software for exact tests and ecumenicism J Hered 1995 86 248 249

[B22] Rousset F GENEPOP’007: a complete re-implementation of the GENEPOP software for Windows and Linux Mol Ecol Resour 2008 8 103 106 10.1111/j.1471-8286.2007.01931.x 21585727

[B23] Weir B Cockerham CC Estimating F-statistics for the analysis of population structure Evolution 1984 38 1358 1370 10.2307/2408641 28563791

[B24] Peakall R Smouse PE GENALEX 6: genetic analysis in Excel. Population genetic software for teaching and research Mol Ecol Notes 2005 6 288 295 10.1093/bioinformatics/bts460 PMC3463245 22820204

[B25] Balloux F Brunner H Lugon‒Moulin N Hausser J Goudet J Microsatellites can be misleading: an empirical and simulation study Evolution 2000 54 1414 1422 11005307 10.1111/j.0014-3820.2000.tb00573.x

[B26] Hedrick PW Perspective: highly variable loci and their interpretation in evolution and conservation Evolution 1999 53 313 318 10.2307/2640768 28565409

[B27] Slatkin M A measure of population subdivision based on microsatellite allele frequencies Genetics 1995 139 457 462 7705646 10.1093/genetics/139.1.457 PMC1206343

[B28] Gaggiotti O Lange O Rassmann K Gliddon C A comparison of two indirect methods for estimating average levels of gene flow using microsatellite data Mol Ecol 2002 8 1513 1520 10564457 10.1046/j.1365-294x.1999.00730.x

[B29] Jost LOU GST and its relatives do not measure differentiation Mol Ecol 2008 17 4015 4026 10.1111/j.1365-294X.2008.03887.x 19238703

[B30] Crawford NG SMOGD: software for the measurement of genetic diversity Mol Ecol Resour 2009 10 556 557 21565057 10.1111/j.1755-0998.2009.02801.x

[B31] Van Oosterhout C Hutchinson WF Wills DPM Shipley P Micro-checker: software for identifying and correcting genotyping errors in microsatellite data Mol Ecol Notes 2004 4 535 538 10.1111/j.1471-8286.2004.00684.x

[B32] Brookfield J A simple new method for estimating null allele frequency from heterozygote deficiency Mol Ecol 1996 5 453 455 8688964 10.1111/j.1365-294x.1996.tb00336.x

[B33] Dieringer D Schlötterer C Microsatellite analyser (MSA): a platform independent analysis tool for large microsatellite data sets Mol Ecol Notes 2003 3 167 169 10.1046/j.1471-8286.2003.00351.x

[B34] Zerlotini A Aguiar ER Yu F Xu H Li Y Young ND Gasser RB Protasio AV Berriman M Roos DS SchistoDB: an updated genome resource for the three key schistosomes of humans Nucl Acids Res 2013 41 D728 D731 10.1093/nar/gks1087 23161692 PMC3531198

[B35] Glenn TC Field guide to next‒generation DNA sequencers Mol Ecol Resour 2011 11 759 769 10.1111/j.1755-0998.2011.03024.x 21592312

[B36] Claesson MJ Wang Q O’Sullivan O Greene-Diniz R Cole JR Ross RP O’Toole PW Comparison of two next-generation sequencing technologies for resolving highly complex microbiota composition using tandem variable 16S rRNA gene regions Nucl Acids Res 2010 38 e200 e200 10.1093/nar/gkq873 20880993 PMC3001100

[B37] Loman NJ Misra RV Dallman TJ Constantinidou C Gharbia SE Wain J Pallen MJ Performance comparison of benchtop high-throughput sequencing platforms Nature Biotech 2012 30 434 439 10.1038/nbt.2198 22522955

[B38] Luo C Tsementzi D Kyrpides N Read T Konstantinidis KT Direct comparisons of Illumina vs. Roche 454 sequencing technologies on the same microbial community DNA sample PLoS One 2012 7 e30087 10.1371/journal.pone.0030087 22347999 PMC3277595

[B39] Mitreva M The genome of a blood fluke associated with human cancer Nature Genet 2012 44 116 118 10.1038/ng.1082 22281765 PMC3718006

[B40] Hauswaldt JS Glenn TC Microsatellite DNA loci from the Diamondback terrapin (*Malaclemys terrapin*) Mol Ecol Notes 2003 3 174 176 10.1046/j.1471-8286.2003.00388.x 15723664

[B41] Steinauer ML Blouin MS Criscione CD Applying evolutionary genetics to schistosome epidemiology Infect Genet Evol 2010 10 433 443 10.1016/j.meegid.2010.02.007 20176142 PMC2861999

[B42] Abbasi I Hamburger J Kariuki C Mungai PL Muchiri EM King CH Differentiating *Schistosoma haematobium* from Related Animal Schistosomes by PCR Amplifying Inter-Repeat Sequences Flanking Newly Selected Repeated Sequences Am J Trop Med Hyg 2012 87 1059 1064 10.4269/ajtmh.2012.12-0243 23109375 PMC3516075

[B43] Zhao G-H Li J Blair D Li X-Y Elsheikha HM Lin R-Q Zou F-C Zhu X-Q Biotechnological advances in the diagnosis, species differentiation and phylogenetic analysis of *Schistosoma* spp Biotech Adv 2012 30 1381 1389 10.1016/j.biotechadv.2012.02.008 22366555

[B44] Valentim CLL LoVerde PT Anderson TJC Criscione CD Efficient genotyping of *Schistosoma manson*i miracidia following whole genome amplification Mol Biochem Parasitol 2009 166 81 84 10.1016/j.molbiopara.2009.02.010 19428677 PMC2771185

[B45] Gower CM Gouvras AN Lamberton PHL Deol A Shrivastava J Mutombo PN Mbuh JV Norton AJ Webster BL Stothard JR Population genetic structure of *Schistosoma mansoni* and *Schistosoma haematobium* from across six sub-Saharan African countries: Implications for epidemiology, evolution and control Acta Trop 2012 doi: 10.1016/j.actatropica.2012.09.014. 10.1016/j.actatropica.2012.09.014 23041540

[B46] Glenn TC Schable NA Isolating microsatellite DNA loci Meth Enzymol 2005 395 202 222 15865969 10.1016/S0076-6879(05)95013-1

[B47] Weber JN Peters MB Tsyusko OV Linnen CR Hagen C Schable NA Tuberville TD McKee AM Lance SL Jones KL Five hundred microsatellite loci for Peromyscus Cons Genet 2010 11 1243 1246 10.1007/s10592-009-9941-x PMC2885811 20563244

[B48] Glenn TC Stephan W Dessauer HC Braun MJ Allelic diversity in alligator microsatellite loci is negatively correlated with GC content of flanking sequences and evolutionary conservation of PCR amplifiability Mol Biol Evol 1996 13 1151 1154 10.1093/oxfordjournals.molbev.a025678 8865669

[B49] Ellegren H Microsatellite mutations in the germline: implications for evolutionary inference Trends Genet 2000 16 551 558 10.1016/S0168-9525(00)02139-9 11102705

[B50] Sheffield VC Weber JL Buetow KH Murray JC Even DA Wiles K Gastier JM Pulido JC Yandava C Sunden SL A collection of tri-and tetranucleotide repeat markers used to generate high quality, high resolution human genome-wide linkage maps Hum Mol Genet 1995 4 1837 1844 10.1093/hmg/4.10.1837 8595404

[B51] Chakraborty R Kimmel M Stivers DN Davison LJ Deka R Relative mutation rates at di-, tri-, and tetranucleotide microsatellite loci Proc Natl Acad Sci U S A 1997 94 1041 1046 10.1073/pnas.94.3.1041 9023379 PMC19636

[B52] Brindley PJ Hotez PJ Break Out: Urogenital Schistosomiasis and *Schistosoma haematobium* Infection in the Post-Genomic Era PLoS Negl Trop Dis 2013 7 e1961 10.1371/journal.pntd.0001961 23556007 PMC3610632

[B53] Webster BL Culverwell CL Khamis I Mohammed KA Rollinson D Stothard JR DNA barcoding of *Schistosoma haematobium* on Zanzibar reveals substantial genetic diversity and two major phylogenetic groups Acta Trop 2012 doi: 10.1016/j.actatropica.2012.06.002. 10.1016/j.actatropica.2012.06.002 22721826

[B54] Webster BL Emery AM Webster JP Gouvras A Garba A Diaw O Seye MM Tchuente LAT Simoonga C Mwanga J Genetic Diversity within *Schistosoma haematobium*: DNA Barcoding Reveals Two Distinct Groups PLoS Negl Trop Dis 2012 6 e1882 10.1371/journal.pntd.0001882 23145200 PMC3493392

[B55] Knopp S Mohammed KA Ali SM Khamis IS Ame SM Albonico M Gouvras A Fenwick A Savioli L Colley DG Study and implementation of urogenital schistosomiasis elimination in Zanzibar (Unguja and Pemba islands) using an integrated multidisciplinary approach BMC Public Health 2012 12 930 10.1186/1471-2458-12-930 23110494 PMC3533998

